# Post-progression outcomes following BCMA-directed CAR T-cell therapy in myeloma: impact of extramedullary and paramedullary disease

**DOI:** 10.3389/fonc.2025.1663814

**Published:** 2025-10-27

**Authors:** Ziad Abuhelwa, Fnu Amisha, Wenyi Fan, Gabriel De Avila, Doris K. Hansen, Ariel F. Grajales-Cruz, Brandon Blue, Omar Castaneda, Hien Liu, Ciara L. Freeman, Taiga Nishihori, Frederick L. Locke, Kenneth H. Shain, Rachid Baz, Melissa Alsina

**Affiliations:** ^1^ H. Lee Moffitt Cancer Center and Research Institute, Tampa, FL, United States; ^2^ Morsani College of Medicine, University of South Florida, Tampa, FL, United States

**Keywords:** multiple myeloma, CAR T-cell therapy, progression, extramedullary disease, survival outcomes

## Abstract

**Introduction:**

BCMA-directed CAR T-cell therapies have improved outcomes in relapsed and refractory multiple myeloma (MM); however, the majority of patients relapse within 1 to 3 years following treatment. Managing disease progression after CAR T-cell therapy remains a major challenge, particularly in aggressive subtypes including extramedullary disease (EMD) and paramedullary disease (PMD). Real-world data on progression patterns post-CAR T cell therapy and the impact of EMD or PMD on outcomes of patients who relapsed post-CAR T-cell therapy remain scarce.

**Methods:**

In this single-center, retrospective study, we evaluated progression patterns and survival outcomes in 106 MM patients who progressed after commercial CAR T-cell therapy (ide-cel or cilta-cel) between May 2021 and December 2023. Overall survival (OS) was defined from the time of post-CAR T-cell therapy progression to death or last follow-up, and progression-free survival (PFS) from post-CAR T-cell therapy progression to progression on the next line of therapy.

**Results:**

Biochemical relapse occurred in 82% of patients, with EMD or PMD present in 51% at progression. Baseline EMD at the time of CAR T-cell infusion was detected in 33% of patients and was associated with significantly inferior PFS (3.6 vs. 7.0 months, p=0.0076) and OS (4.8 vs. 21.0 months, p=0.00086) compared to those without EMD. Similarly, the presence of EMD at progression was associated with shorter PFS (4.7 vs. 8.5 months, p=0.022) and OS (7.4 vs. 21.1 months, p=0.035). Patients who were EMD positive at both baseline and progression had the poorest outcomes. PMD at baseline or progression was not significantly associated with worse survival.

**Discussion:**

Our findings highlight that post-CAR T-cell progression in MM is heterogeneous and that EMD confers an adverse prognosis, emphasizing the critical need for imaging surveillance. Strategies such as bridging therapies aimed at reducing tumor burden prior to CAR T-cell infusion or maintenance therapies post-CAR T-cell therapy warrant further investigation to optimize responses and improve long-term survival in this high-risk population.

## Introduction

Multiple myeloma (MM) makes up 1.8% of all new cancer cases in the United States, and it is characterized by a remitting relapsing course ultimately leading to refractoriness to treatment (1). Patients with MM typically experience disease progression either biochemically or symptomatically with end-organ damage and extramedullary diseases (EMD) or paramedullary disease (PMD) (2). EMD is characterized by soft tissue plasmacytomas resulting from hematogenous spread of malignant cells outside the bone marrow, while PMD occurs due to direct tumor extension from skeletal lesions following cortical bone disruption (3). EMD and PMD are uncommon at initial diagnosis but occurs more frequently at the time relapse or disease progression (3–5). Patients with EMD or PMD at time of diagnosis or progression tend to have worse outcomes than those without (6–9).

Idecabtagene vicleucel (ide-cel) and ciltacabtagene autoleucel (cilta-cel) are B-cell maturation antigen (BCMA)-directed chimeric antigen receptor T-cell (CAR T-cell) therapies that have demonstrated significant efficacy in patients with heavily pretreated MM patients, as shown in the pivotal KarMMa-1 and CARTITUDE-1 trials, respectively (10, 11). Subsequently, the phase 3 KarMMa-3 and CARTITUDE-4 trials evaluated the use of ide-cel and cilta-cel in earlier lines of therapy, demonstrating superior progression-free survival (PFS) compared to standard regimens in patients with relapsed and refractory MM (12, 13). Despite these advances, relapse after CAR-T-cell therapies remains a major challenge, often presenting in aggressive forms such as EMD or PMD, which are associated with particularly poor outcomes.

The detailed knowledge of patterns of progression after BCMA directed CAR T-cell therapy is lacking in the literature. Understanding how patients progress and how this affects prognosis is important to developing optimal disease monitoring strategies and interventions. Our study aims to assess the patterns of progression after commercial BCMA directed CAR T-cell therapy and to assess the impact of the presence of EMD and PMD at baseline prior to CAR T-cell therapy and at time of progression on the survival outcomes.

## Methods

### Study design and settings

This is a single-center retrospective study conducted at the H. Lee Moffitt Cancer Center and Research Institute in Tampa, Florida, with prior approval from the institutional review board.

### Patients

All adult patients (≥18 years) with MM who received commercial BCMA-directed CAR T-cell therapy (ide-cel or cilta-cel) between May 1, 2021, and December 31, 2023, and subsequently experienced disease progression were included in the study. At our institution, baseline laboratory tests, imaging, and bone marrow assessments are performed on all MM patients prior to CAR T-cell infusion. On day 90 post-CAR T-cell therapy, repeat laboratory tests, imaging (positron emission tomography, computed tomography, or whole-body magnetic resonance imaging), and bone marrow assessments are conducted to evaluate treatment response. Subsequently patients are monitored per standard of care with the treating physicians with monitoring of serum and urine paraprotein as well as needed imaging tests. In the event of suspected disease progression, a restaging work up with imaging and bone marrow biopsy is performed.

### Data collection and clinical assessment

Laboratory, imaging, and bone marrow data were collected at three time points: baseline prior or at the time of CAR T-cell infusion, at day 90 post-infusion, and at disease progression. Response and progression were evaluated using the International Myeloma Working Group (IMWG) criteria (14). Biochemical relapse is defined as a 25% or greater increase from the lowest response level in any of the following markers: serum M-protein (with an absolute increase ≥ 0.5 g/dL), urine M-protein (with an absolute increase ≥ 200 mg/24h), or the difference between involved and uninvolved free light chain (FLC) levels in patients without measurable serum or urine M-protein (with an absolute increase in FLC ≥ 10 mg/dL) with or without evidence of end-organ damage. Biopsy confirmation was not universally required to confirm EMD or PMD, and they were identified through imaging with EMD showing myeloma involvement in soft tissues and all soft tissue lesions were reviewed and non-contiguous with bone, disease and PMD characterized by direct tumor extension from skeletal lesions. Longitudinal assessment was used retrospectively to increase confidence in the diagnosis of EMD and PMD. At the time of progression post-CAR T-cell therapy, bone marrow involvement was defined by the presence of clonal plasma cells at or above 5%. High-risk cytogenetics were defined as the presence of del(17p)/monosomy 17, t(4,14), or t(14,16) on fluorescence *in situ* hybridization testing. Overall survival (OS) was defined as the time from progression after CAR T-cell therapy to death from any cause or last follow-up. PFS was defined as the time from progression after CAR T-cell therapy to progression on the earliest subsequent therapy.

### Statistical analysis

The distribution of progression patterns was analyzed overall, as well as based on the presence of any EMD or PMD and the type of CAR T-cell therapy using the chi-square test for categorical variables and independent t-test for continuous variables. Kaplan-Meier survival curves were used to estimate PFS and OS, with log-rank tests applied to compare survival outcomes across selected variables, including baseline and progression EMD or PMD status and the type of CAR T-cell therapy. A p-value of <0.05 was considered statistically significant. All statistical analyses were performed using R program version 3.5.1.

## Results

**Figure 1:**
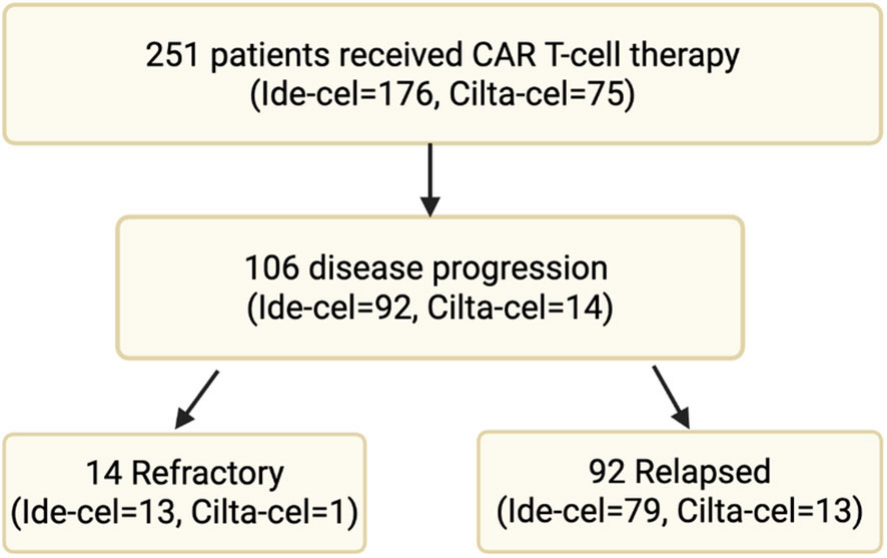
Multiple myeloma patients received commercial anti-BCMA CAR T-cell therapy.

A total of 251 patients with MM received commercial anti-BCMA CAR T-cell therapy: either ide-cel (n=176) or cilta-cel (n=75) between May 2021 and December 2023. Of them, 106 patients (ide-cel n=92, cilta-cel n=14) had disease progression at the time of data cut off and were included in the study. Among them, 14 patients (ide-cel n=13 and cilta-cel n=1) had refractory disease, defined as progression within 60 days of CAR T-cell infusion. The remaining 92 patients (ide-cel n=79, cilta-cel n=13) had relapsed disease, with progression occurring beyond 60 days post-infusion (**Figure 1**).

### Baseline characteristics

The median age of the included patients was 66 years (Interquartile Range (IQR): 58-73), with the majority being male (n=59, 56%) and white (n=70, 66%). IgG was the most common heavy chain involved, seen in 60 patients (57%), while kappa was the predominant light chain in 71 patients (67%). Oligo- or non-secretory disease was observed in 4 patients (4%). Anemia (hemoglobin < 10 g/dL) prior to CAR T-cell infusion was present in 58 patients (55%), which was more frequent than neutropenia (absolute neutrophil count < 1000/mL) in 11 patients (10%) and thrombocytopenia (platelets count < 100 x 10^9^/L) in 37 patients (35%). The median number of prior lines of treatment was 6 (IQR: 5-7), and most patients (n=84, 79%) had undergone a prior autologous stem cell transplant. High-risk cytogenetics were found in 38% of patients (n=40), with del(17p) present in 30% (n=31), t(4,14) in 13% (n=13) and t(14,16) in 6% (n=6). By day 90 post-CAR T-cell therapy, 67% of patients had achieved an objective-response rate based on IMWG response criteria with statistically lower response in patients with EMD compared with those without EMD (47% vs 72%, p=0.01). Otherwise, baseline characteristics were well balanced, with no statistically significant differences between patients with and without EMD (**Table 1**).

**Table 1:** Baseline characteristics of the included patients.

Characteristic		All patients (n=106)	Any EMD (n=35)	No EMD (n=71)	p-value
Median age in years (IQR)		66 (58–73)	63 (58–69)	68 (58–74)	0.10
Male, n (%)		59 (56%)	20 (57%)	39 (55%)	0.83
Race, n (%)	White	70 (66%)	21 (60%)	49 (69%)	0.72
	African American	15 (14%)	6 (17%)	9 (13%)	
	Hispanic	17 (16%)	6 (17%)	11 (15%)	
	Other	4 (4%)	2 (6%)	2 (3%)	
ECOG PS, n (%)	0-1	94 (89%)	30 (86%)	64 (90%)	0.52
	2	10 (9%)	4 (11%)	6 (9%)	
	≥ 3	2 (2%)	1 (3%)	1 (1%)	
Heavy chain monoclonal component, n (%)	IgG	60 (57%)	24 (69%)	36 (51%)	0.05
	IgA	12 (11%)	1 (3%)	11 (15%)	
	IgD	1 (1%)	1 (3%)	0 (0)	
	None	33 (31%)	9 (26%)	24 (34%)	
Light chain monoclonal component, n (%)	Kappa	71 (67%)	24 (69%)	47 (66%)	0.99
	Lambda	31 (29%)	10 (29%)	21 (30%)	
	None	4 (4%)	1 (3%)	3 (4%)	
Non- or oligosecretory, n (%)		4 (4%)	1 (3%)	3 (4%)	0.99
Complete blood count	Anemia*, n (%)	58 (55%)	19 (54%)	39 (55%)	0.95
	Neutropenia†, n (%)	11 (10%)	4 (11%)	7 (9.9%)	0.99
	Thrombocytopenia^+^, n (%)	37 (35%)	13 (37%)	24 (34%)	0.73
Renal failure‡, n (%)		4 (4%)	1 (3%)	3 (4%)	0.99
Median number of prior lines of therapy (IQR)		6 (5–7)	5 (4–7)	6 (5–7)	0.11
Prior Auto-SCT, n (%)		79 (75%)	28 (80%)	51 (73%)	0.42
High risk cytogeneticˆ, n (%)		40 (38%)	18 (55%)	47 (68%)	0.18
del(17p)/monosomy17, n (%)		31 (30%)	7 (21%)	24 (35%)	0.16
t(4,14), n (%)		13 (13%)	4 (12%)	9 (13%)	0.99
t(14,16), n (%)		6 (6%)	1 (3%)	5 (7%)	0.66
Chromosome 1 abnormalities, n (%)		59 (58%)	15 (47%)	44 (64%)	0.11
t(11,14), n (%)		17 (17%)	7 (22%)	10 (14%)	0.36
Hyperdiploidy, n (%)		28 (28%)	8 (25%)	20 (29%)	0.68
PMD		18 (17%)	7 (20%)	11 (15%)	0.56
Day-90 response, n (%)	ORR	71 (67%)	16 (47%)	50 (72%)	0.01
	sCR/CR	53 (50%)	14 (40%)	39 (55%)	0.15

IQR, interquartile range; ECOG PS, Eastern Cooperative Oncology Group performance status; SCT, stem cell transplant; R-ISS, revised international staging system; EMD, extramedullary disease; PMD, paramedullary disease; ORR, objective response rate; sCR/CR, stringent complete response/complete response.

*Hemoglobin <10 g/dL.

†Absolute neutrophil count < 1000/mL.

^+^Platelets count < 100 x 10^9^/L.

‡Serum creatinine > 2 mg/dL.

ˆPresence of del(17p)/monosomy 17, t(4,14), or t(14,16) on fluorescence *in situ* hybridization testing.

### Patterns of progression

The median time to progression in this cohort of patients was 7.7 months (IQR: 3.2–11.5 months). Biochemical recurrence with or without symptomatic disease was the most frequent pattern of relapse, occurring in 82% of patients (n=83), followed by bone marrow involvement in 59% (n=51), EMD or PMD in 51% (n=51), and new bone lesions in 50% (n=47). Isolated sites of relapse were relatively uncommon, with isolated biochemical progression seen in 6% (n=6), isolated EMD or PMD in 5% (n=5), isolated bone marrow involvement in 0% (n=0), and isolated new bone disease in 2% (n=2) (**Table 2**). There were no significant differences in progression patterns between patients treated with ide-cel and those treated with cilta-cel.

**Table 2:** Patterns of disease progression after CAR T-cell therapy.

Progression pattern	All patients (N = 106)
Median time to progression mo (IQR)	7.7 (3.2-11.5)
Biochemical recurrence, n (%)	83 (82%)
BM involvement, n (%)	51 (59%)
New bone disease, n (%)	47 (50%)
EMD or PMD, n (%)	51 (51%)
Any EMD^, n (%)	45 (45%)
Any PMD^^, n (%)	9 (9%)
Isolated BM involvement, n (%)	0 (0%)
Isolated new bone disease, n (%)	2 (2%)
Isolated biochemical progression, n (%)	6 (6%)
Isolated EMD or PMD, n (%)	5 (5%)

Mo, months; IQR, interquartile range; BM, bone marrow; EMD, extramedullary disease; PMD, paramedullary disease.

^ with or without PMD.

^^ with or without EMD.

After progression on CAR T-cell therapy, conventional combination regimens were most frequently used (48%) line of therapy, followed by BCMA-directed (30%) and GPRC5D-directed bispecifics (8%). Treatment patterns were comparable between patients with and without EMD, with no statistically significant differences observed (**Table 3**).

**Table 3:** First line therapies after disease progression.

Therapy	All patients (n=106)	Any EMD (n=35)	No EMD (n=71)	p-value
BCMA bispecific therapy	29 (30%)	9 (32%)	20 (29%)	0.19
GPRC5D bispecific therapy	8 (8%)	1 (4%)	7 (10%)	
Conventional combination therapies	46 (48%)	13 (46%)	33 (49%)	
Clinical trial	2 (2%)	2 (7%)	0 (0%)	
Other BCMA targeted therapy	4 (4%)	0 (0%)	4 (6%)	
Hospice	7 (7%)	3 (11%)	4 (6%)	
Unknown	10	7	3	

BCMA, b-cell maturation antigen; GPRC5D, G protein coupled receptor, class C, group 5, member D.

### EMD and PMD

**Figure 2:**
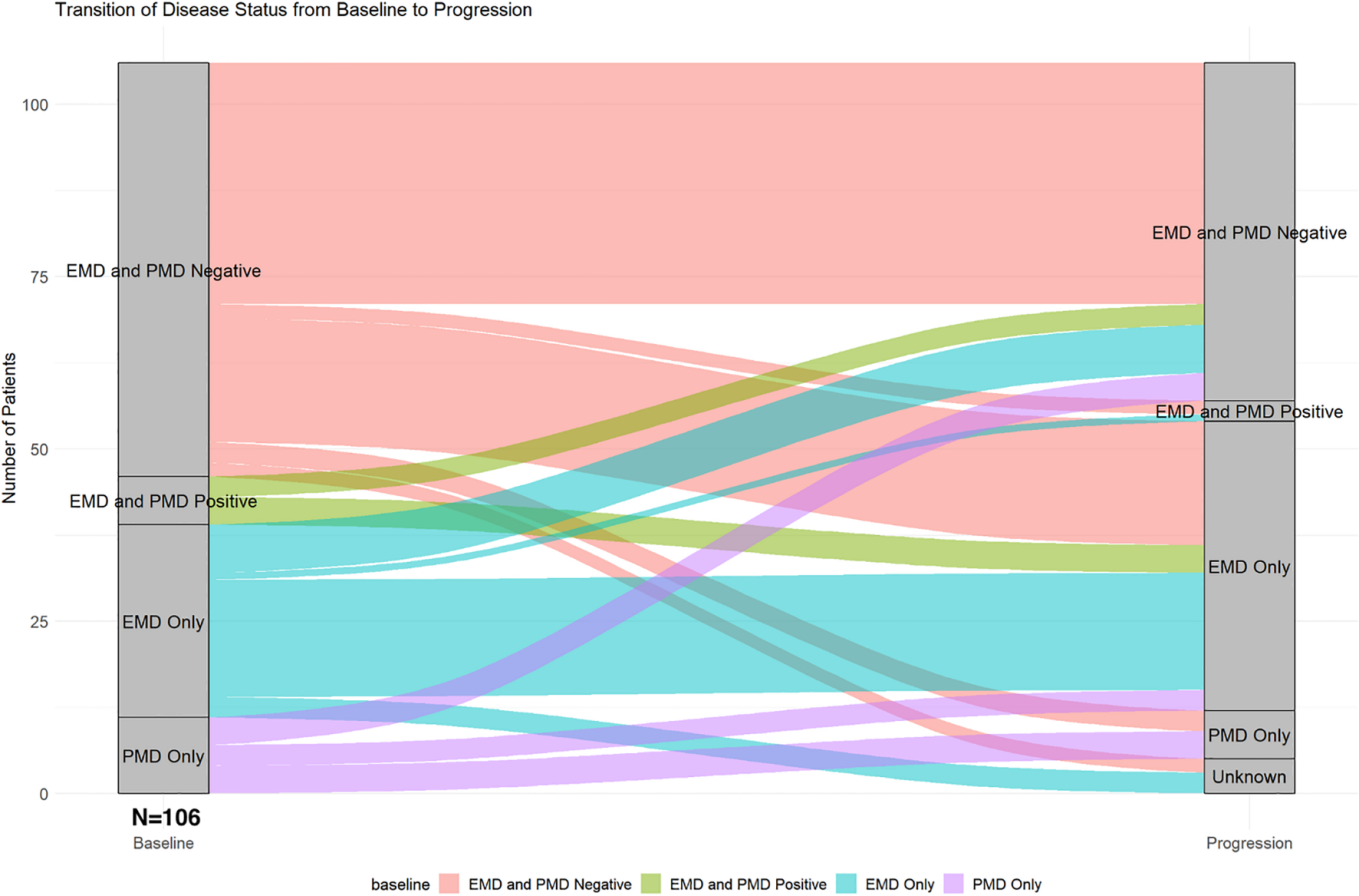
Transition of extramedullary and paramedullary disease status from baseline to progression.

Baseline EMD or PMD at the time of CAR T-cell infusion was detected in 46 patients (43%), including 35 patients (33%) with any EMD (with or without PMD) and 18 patients (17%) with any PMD (with or without EMD). The most common sites of EMD/PMD were skin and soft tissue in 13 patients (13%), followed by lymph nodes and paraspinal masses in 9 patients (9%) each. At progression, similar patterns were observed: skin and soft tissue in 16 patients (17%), lymph nodes in 13 (17%), and lung/pleura in 14 (15%). Patients with baseline EMD were more likely to have EMD at progression (p=0.035); similarly, those with baseline PMD were more likely to have PMD at progression (p=0.049). **Figure 2** shows the transition of EMD and PMD status from baseline at the time of CAR T-cell infusion to time of progression. Among 60 patients (57%) who were EMD- and PMD-negative at baseline, 35 (58%) remained negative, 18 (30%) developed EMD only, 3 (5%) developed PMD only, 2 (3%) developed both EMD and PMD, and 2 (3%) had unknown status at progression. Of the 7 patients (7%) with both EMD and PMD at baseline, 4 (57%) transitioned to EMD only, and 3 (43%) became EMD- and PMD-negative. Among 28 patients (26%) with EMD only at baseline, 17 (61%) remained unchanged, 1 (4%) progressed to both EMD and PMD, 7 (25%) became negative, and 3 (11%) had unknown progression status. Of the 11 patients (10%) with PMD only, 4 (36%) remained in the same category, 3 (27%) transitioned to EMD only, and 3 (27%) became negative for both EMD and PMD.

### Survival data

**Figure 3:**
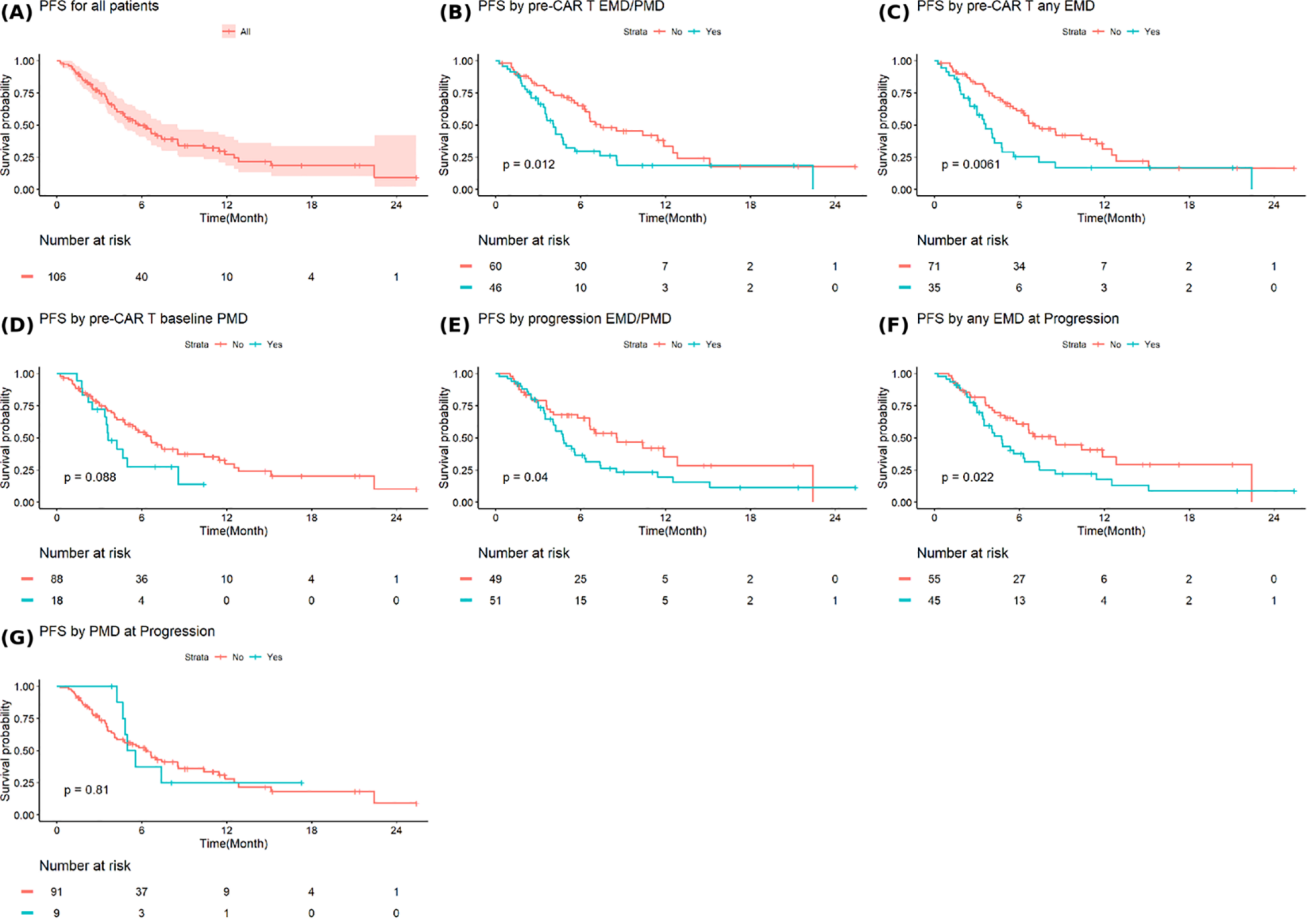
Progression free survival in **(A)** all patients and based on **(B)** extramedullary or paramedullary disease (EMD or PMD) at baseline **(C)** any EMD at baseline **(D)** any PMD at baseline **(E)** EMD or PMD at progression **(F)** any EMD at progression **(G)** any PMD at progression. * Progression free survival (PFS2) is defined as the time from progression after CAR T-cell therapy to progression on the earliest subsequent therapy.

The median PFS after progression from CAR T-cell therapy to progression on the earliest subsequent therapy for all patients was 5.8 months (95% Confidence Interval [CI]: 4.7-8.5) (**Figure 3A**). Patients with baseline EMD or PMD had significantly shorter median PFS after progression from CAR-T cell therapy compared to those without (4.1 months (95%CI: 3.5-5.6) vs. 7.4 months (95%CI: 6.4-12.8), p=0.012) (**Figure 3B**). Similarly, patients with any baseline EMD had a shorter median PFS 3.6 months (95%CI: 3.0-5.6) versus 7.0 months (95%CI: 6.3-12.5) for those without any baseline EMD (p=0.0061) (**Figure 3C**). Baseline any PMD was not associated with a significant difference in PFS (**Figure 3D**). Patients with EMD or PMD at progression post-CAR T-cell therapy had a median PFS of 4.8 months (95% CI: 4.1-6.4), significantly shorter than 8.6 months (95% CI: 6.6-NA) in those without (p=0.04) (**Figure 3E**). The presence of any EMD at progression was also associated with inferior PFS (4.7 months (95% CI: 3.5-7.4) vs. 8.5 months (95% CI: 5.8-NA), p=0.022) (**Figure 3F**). Any PMD at progression did not significantly impact PFS (**Figure 3G**).

**Figure 4:**
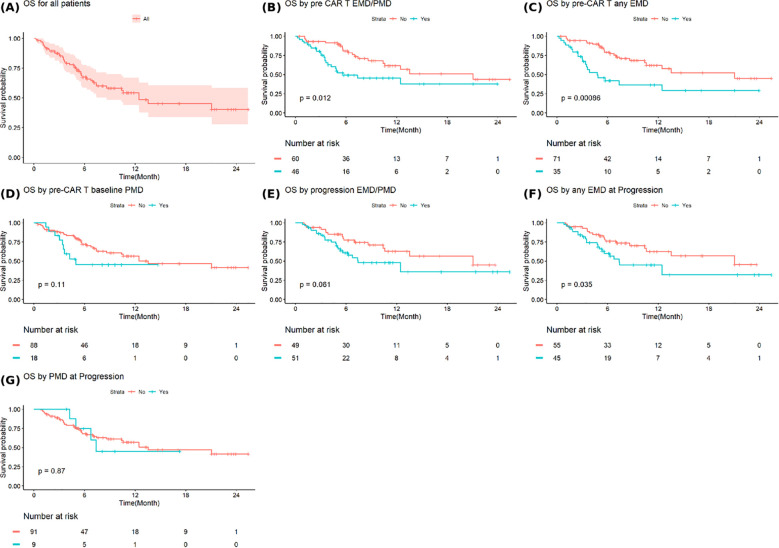
Overall survival in **(A)** all patients and based on **(B)** extramedullary or paramedullary disease (EMD or PMD) at baseline **(C)** any EMD at baseline **(D)** any PMD at baseline **(E)** EMD or PMD at progression **(F)** any EMD at progression **(G)** any PMD at progression. * Overall survival is defined as the time from progression after CAR T-cell therapy to death from any cause or last follow-up.

The median OS after progression from CAR T-cell therapy for all patients was 12.5 months (95%CI: 8.6-NA) (**Figure 4A**). Patients with baseline EMD or PMD had a significantly shorter median OS of 5.7 months (95% CI: 3.9-NA) compared to 21.1 months (95% CI: 10.6-NA) in those without baseline involvement (p=0.012) (**Figure 4B**). Similarly, patients with baseline any EMD experienced inferior median OS of 4.8 months (95% CI: 3.4–NA) versus 21.1 months (95% CI: 10.6–NA) among those without EMD (p=0.00086) (**Figure 4C**). Baseline any PMD was not associated with a significant difference in OS (**Figure 4D**). Patient with EMD or PMD at progression post-CAR T-cell therapy continued to show worse outcomes, with a median OS of 7.4 months (95% CI: 5.6-NA) compared to 21.1 months (95% CI: 10.6-NA) in those without, although this did not reach statistical significance (p=0.061) (**Figure 4E**). However, the presence of any EMD at progression was significantly associated with shorter OS (7.4 months (95% CI: 5.4-NA) vs. 21.1 months (95% CI: 10.6-NA), p=0.035) (**Figure 4F**). Any PMD at progression did not significantly affect OS (**Figure 4G**).

**Figure 5:**
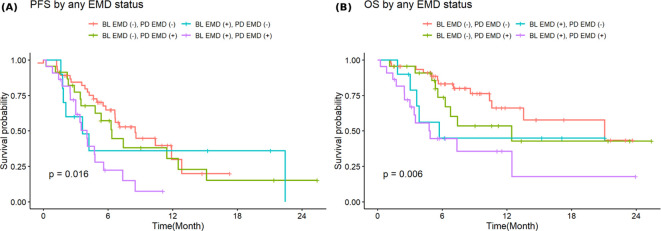
Stratified progression-free **(A)** and overall survival **(B)** by combined baseline and progression extramedullary disease.

**Figure 5** shows PFS and OS stratified by both baseline and progression any EMD status. Patients with EMD present at both baseline and progression had the worst outcomes, post-CAR T-cell therapy progression, with a median PFS of 4 months (95%CI: 3-7.4; p=0.016) and a median OS of 4.9 months (95%CI: 3.0-NA, p*=*0.006) compared to other groups. In contrast, patients who were negative for EMD at baseline and progression experienced the most favorable outcomes post-CAR T-cell therapy progression with a median PFS of 8.6 months (95%CI: 6.6-NA) and a median OS of 21.0 months (95%CI: 13.6-NA).

## Discussion

This single-center, retrospective real-world analysis characterizes the patterns of disease progression following standard-of-care CAR T-cell therapy in patients with MM, with a specific focus on the impact of EMD and PMD on survival outcomes. Our findings demonstrate that post-CAR T-cell therapy progression is heterogeneous, with the majority of patients experiencing biochemical relapse, but is associated with a high rate of EMD and PMD. While about 5% of patient had EMD or PMD as the sole evidence of progression and an additional 2% progressed solely with new bone disease, these findings highlight the importance of imaging in the follow up and monitoring of patients with multiple myeloma post-BCMA directed CAR T-cell therapy. Notably, none of the patients in our cohort exhibited isolated bone marrow involvement at the time of progression, suggesting that routine bone marrow monitoring following BCMA-directed CAR T-cell therapy, in the absence of other signs of progression, have limited clinical utility to detect isolated marrow relapse.

Our study highlights that the presence of EMD, at baseline or at the time of progression, is associated with inferior PFS and OS after progression post-CAR T-cell therapy, reinforcing the prognostic significance of this disease feature. While the survival outcomes of patients with PMD alone is numerically inferior to those without, the difference did not reach statistical significance likely reflecting the limited sample size and this warrants further investigation. Although patients with baseline EMD or PMD are more likely to exhibit EMD or PMD at progression, the absence of these features at baseline does not eliminate their emergence at progression, underscoring the importance of imaging monitoring after CAR T-cell therapy and at the time of disease progression.

With advancements in novel therapeutic agents and improved survival in MM, the incidence of EMD has become increasingly common, particularly at the time of relapse. The pathophysiology underlying EMD is multifactorial and may involve heightened systemic inflammation and a more immunosuppressive tumor microenvironment. These features are often associated with high tumor burden and increased metabolic activity, as visualized on positron emission tomography imaging. Additionally, EMD is frequently characterized by bulky, metabolically active lesions that impair T-cell functionality, both in the collected autologous T cells used for CAR T-cell manufacturing and in the infused CAR T-cells. This contributes to a mismatch between effector cells and tumor burden, ultimately requiring robust CAR T-cell expansion to achieve meaningful and sustained responses. Notably, high metabolic tumor volume has been linked to both increased toxicity and reduced efficacy of BCMA-directed CAR T-cell therapy in MM (15–20).

In the KarMMa-1 trial in a heavily pretreated cohort, EMD, defined as soft-tissue lesions not contiguous with bone, was observed in 39% of patients, with ide-cel maintaining the objective response rate in this high-risk subgroup (10). In KarMMa-3, in patients with 2–4 prior lines, EMD was more broadly defined to include both true extramedullary soft-tissue disease and bone-related soft-tissue plasmacytomas, with an incidence of 24%, and its presence correlated with significantly shorter PFS (21). Similarly, the CARTITUDE-1 trial reported EMD, including both bone-based and extramedullary plasmacytomas, in 20% of patients, where it was associated with reduced PFS (22). In the CARTITUDE-4 trial, 21.2% of patients had EMD, which was likewise linked to inferior PFS and OS outcomes (13). Real-world experiences have reported higher incidence of EMD compared to clinical trials. For example, in a study of 159 patients treated with ide-cel, 48% had either a history of or active EMD at the time of infusion (23). Another multicenter analysis involving 152 patients who received commercial CAR T-cell therapy found that 31% had active EMD at infusion (24). Both studies demonstrated that the presence of EMD was associated with inferior response rates and survival outcomes.

Overall, our findings align with existing literature demonstrating inferior outcomes in patients with EMD. The high incidence of EMD in our study is likely due to the heavily pretreated nature of our cohort as the risk of developing EMD increases with cumulative treatment exposure and disease refractoriness (25). Some studies define EMD strictly as hematogenous spread without adjacent bone involvement, while others include both EMD and PMD. These inconsistencies in classification highlight the need for caution when interpreting and comparing results across studies.

Our study demonstrated that the presence of EMD at the time of progression after CAR T-cell therapy was associated with inferior survival outcomes. While most published studies have focused on the prognostic impact of EMD prior to CAR T-cell infusion, our findings highlight the significance of disease characteristics at progression. Notably, patients with baseline EMD were more likely to have EMD at progression, and this ultra-high-risk group experienced significantly worse survival outcomes, underscoring the aggressive and persistent nature of this disease phenotype, even after CAR T-cell therapy.

To improve outcomes in patients with EMD undergoing standard-of-care CAR T-cell therapy, there is a critical need to establish standardized treatment protocols tailored to this high-risk population. Bridging therapies designed to reduce tumor burden prior to CAR T-cell infusion may be beneficial in enhancing treatment efficacy. Among these, radiation therapy has shown promise as a localized bridging strategy, effectively reducing bulky disease without significantly increasing toxicity, and potentially improving CAR T-cell expansion and response (24, 26). However, data on the optimal type, timing, and integration of bridging therapies remain limited. Maintenance therapy following CAR T-cell therapy, including the use of lenalidomide or novel bispecific antibodies, is currently under investigation and may improve the durability of response, particularly in high-risk patient populations (27–29). Prospective studies are warranted to better define the role of these interventions and optimize outcomes in this challenging subgroup.

Limitations of our study include the relatively small sample size and the predominance of patients treated with ide-cel, with a minority having progressed after receiving cilta-cel. Response assessments were conducted at the discretion of the treating physician, without independent review, which may introduce variability. Additionally, EMD and PMD was identified solely through radiographic imaging without histopathologic confirmation. Although imaging is essential for detecting EMD and PMD, relying exclusively on radiologic findings may overestimate its prevalence, as false positives are possible in the absence of biopsy-proven disease. Despite these limitations, our study offers meaningful insights into real-world patterns of progression following commercial CAR T-cell therapy and demonstrates that the presence of EMD is associated with poor outcomes, underscoring the need for targeted strategies to improve prognosis in this high-risk population.

## Conclusion

In this real-world retrospective analysis, we characterized patterns of disease progression following commercial anti-BCMA CAR T-cell therapy and demonstrated that the presence of EMD, both at baseline and at the time of progression, is associated with significantly inferior survival outcomes after progression post-CAR T-cell therapy. Our findings reinforce the aggressive and refractory nature of EMD and highlight that patients with these high-risk features are more likely to progress early and die sooner than those without such involvement. This underscores the clinical relevance of assessing and monitoring for EMD and PMD throughout the treatment course of patients undergoing CAR T-cell therapy.

Given the poor prognosis associated with EMD, there is a critical need for therapeutic strategies to improve outcomes in this high-risk subgroup. Bridging therapies aimed at reducing tumor burden prior to CAR T-cell infusion may enhance CAR T-cell expansion, persistence, and antitumor activity. Additionally, maintenance therapies post-infusion may prolong response durability. However, the optimal approach to bridging and maintenance remains undefined and warrants prospective investigation.

## Data Availability

The raw data supporting the conclusions of this article will be made available by the authors, without undue reservation.
